# A randomized controlled trial to compare the effects of time-restricted eating versus Mediterranean diet on symptoms and quality of life in bipolar disorder

**DOI:** 10.1186/s12888-024-05790-4

**Published:** 2024-05-18

**Authors:** Sheri L. Johnson, Greg Murray, Lance J. Kriegsfeld, Emily N.C. Manoogian, Liam Mason, J. D. Allen, Michael Berk, Satchidanda Panda, Nandini A. Rajgopal, Jake C. Gibson, Keanan J. Joyner, Robert Villanueva, Erin E. Michalak

**Affiliations:** 1 Department of Psychology, University of California, Berkeley, USA; 2 Centre for Mental Health, Swinburne University, Melbourne, VIC 3122 Australia; 3 Regulatory Biology, Salk Institute for Biological Studies, La Jolla, CA 92037 USA; 4 Research Department of Clinical, Educational and Health Psychology, University College London, London, UK; 5 School of Medicine, Deakin University, Geelong, VIC Australia; 6 Department of Psychiatry, University of British Columbia, Vancouver, BC Canada

**Keywords:** Bipolar disorder, Randomized controlled trial, Time-restricted eating, Mediterranean diet, Quality of life, Depression, Mania, Circadian

## Abstract

**Background:**

The primary objective of this randomized controlled trial (RCT) is to establish the effectiveness of time-restricted eating (TRE) compared with the Mediterranean diet for people with bipolar disorder (BD) who have symptoms of sleep disorders or circadian rhythm sleep–wake disruption. This work builds on the growing evidence that TRE has benefits for improving circadian rhythms. TRE and Mediterranean diet guidance will be offered remotely using self-help materials and an app, with coaching support.

**Methods:**

This study is an international RCT to compare the effectiveness of TRE and the Mediterranean diet. Three hundred participants will be recruited primarily via social media. Main inclusion criteria are: receiving treatment for a diagnosis of BD I or II (confirmed via DIAMOND structured diagnostic interview), endorsement of sleep or circadian problems, self-reported eating window of ≥ 12 h, and no current mood episode, acute suicidality, eating disorder, psychosis, alcohol or substance use disorder, or other health conditions that would interfere with or limit the safety of following the dietary guidance. Participants will be asked to complete baseline daily food logging for two weeks and then will be randomly allocated to follow TRE or the Mediterranean diet for 8 weeks, during which time, they will continue to complete daily food logging. Intervention content will be delivered via an app. Symptom severity interviews will be conducted at baseline; mid-intervention (4 weeks after the intervention begins); end of intervention; and at 6, 9, and 15 months post-baseline by phone or videoconference. Self-rated symptom severity and quality of life data will be gathered at those timepoints, as well as at 16 weeks post baseline. To provide a more refined index of whether TRE successfully decreases emotional lability and improves sleep, participants will be asked to complete a sleep diary (core CSD) each morning and complete six mood assessments per day for eight days at baseline and again at mid-intervention.

**Discussion:**

The planned research will provide novel and important information on whether TRE is more beneficial than the Mediterranean diet for reducing mood symptoms and improving quality of life in individuals with BD who also experience sleep or circadian problems.

**Trial registration:**

ClinicalTrials.gov ID NCT06188754.

## Background

Bipolar disorder (BD) is the fifth leading cause of disability worldwide according to the World Health Organization [1]. Individuals with BD experience disproportionately high unemployment rates, legal difficulties, lower educational accomplishment, and profound functional impairment [1–5]. Furthermore, a chronic and sustained illness course, including frequent relapse and hospitalization, is normative for those with BD even with the best empirically supported treatment [6, 7].

The current study builds on two lines of evidence. First, a growing body of work indicates that time-restricted eating (TRE), in which eating is limited to 6–10 h during wake periods, improves diurnal rhythms of clock gene expression, cortisol, and glucose [8–13]. Second, extensive research indicates that diurnal rhythms of sleep/wake and activity are often disrupted in patients with BD and can trigger or worsen symptoms [14].

## Time-Restricted Eating (TRE)

Animal and human science show that the timing of food consumption powerfully influences diurnal rhythms. In groundbreaking work on this topic, researchers experimentally randomized mice to time-restricted feeding during wake hours vs. 24 h of ad libitum feeding. The caloric intake and other facets of diet were yoked for strict control. Time-restricted feeding (TRF) had powerful benefits for molecular rhythms in circadian clock components, and improvements in glucose tolerance and insulin resistance have been well replicated across studies [9–11, 15–18]; moreover, recent data show that it is beneficial for animal longevity independent of and complementary to caloric restriction [19]. Mechanistic data suggest that, by coordinating peripheral oscillators, TRF helps coordinate diurnal rhythms between brain cells in the suprachiasmatic nucleus and cells in other organs, e.g., the heart, lung, kidneys, and adrenal glands [20, 21]. Indeed, converging evidence indicates that at least some metabolic benefits of TRF relate to the restoration of circadian rhythms [20].

Drawing on these impressive findings in animals, researchers have examined the benefits of time-restricted eating (TRE) for humans. Across at least 14 randomized controlled trials on human physical and medical outcomes, including several with large sample sizes [22], benefits have been demonstrated for glucose regulation and, in many studies, decreased body mass index. In a randomized controlled trial of 80 healthy males, TRE was shown to lead to enhanced circadian gene expression, as indicated by the upregulation of BMAL1, CLOCK, and SIRT1 [23]. Two small studies have shown TRE-related shifts in the circadian rhythm of clock gene expression [12, 13].

Participants assigned to TRE reported high satisfaction and high adherence [20, 24]. This high adherence may stem from its subjective benefits. Within a few weeks, participants reported improved energy and restedness, which were sustained for at least six months postintervention [13, 24]. Several studies using sleep logs have shown that TREs enhance perceived restfulness and subjective sleep quality [13, 24, 25]. Relatedly, a single study using actigraphy-based sleep indicators indicated improved sleep duration and efficiency and diminished variability in sleep timing after 10 h of TRE for 12 weeks [24]. Across studies, quality of life ratings significantly improved from baseline to the 6-month follow-up among participants assigned to TRE [25–28]. Moreover, TRE does not increase hunger [20, 29], harm muscle mass [30], or adversely impact cognitive performance [31].

## Background on Bipolar Disorder (BD)

Biological rhythm disturbance is one of the major etiological models of BD [32]. Disturbed sleep-activity rhythms are core symptoms of manic and depressive episodes [33, 34], and these disturbances persist at milder levels for most people with BD during remission [35–38]. Evidence from naturalistic studies of sleep, jet lag, activity, and sleep deprivation indicates that sleep disturbances predict BD onset and symptom increases [14, 38–42]. These issues also appear tied to vulnerability to the disorder, given evidence linking (a) BD to polymorphisms of multiple circadian genes, (b) mood variability to polygenic risk scores related to chronotype and low circadian amplitude, and (c) familial risk for BD to disruptions in sleep/sleep-activity rhythms [43–45]. Accordingly, there is an urgent need for interventions that can stabilize biological rhythms in patients with BD.

### Biological rhythm intervention in BD

Psychopharmacological and psychotherapeutic treatments for BD have well-documented effects on circadian function. Animal research provides considerable evidence that lithium and antidepressants influence biological mechanisms involved in regulating circadian rhythms [46–50]. Parallel work in humans suggests that changes in neuronal circadian rhythms predict lithium response [51] and that psychosocial treatments for mood disorders decrease REM density [52–54]. Pilot research has shown the benefits of pharmacological agents such as ramelteon and agomelatine, which have selective affinities for the melatonin receptors MT1 and MT2, as adjuncts for mood stabilizers in BD [55, 56]

Multiple behavioral treatments for BD, including interpersonal and social rhythm therapy (IPSRT; [57]), light therapy [58], and interventions designed to restrict activity during dark periods [59, 60], focus on improving sleep and circadian function. Treatments designed to address sleep and circadian rhythms in BD have not been panaceas. Dark therapy, which may require years to successfully implement [59], appeared to help inpatients only when applied early in manic episodes [60]. IPSRT attempts to change the regularity of many different activities, and perhaps due to this complexity, many IPSRT patients are not successful at changing overall rhythms [61], which could explain the mixed findings on its effectiveness relative to control conditions [62, 63]. CBT for insomnia appears helpful for those with BD but does not target broader circadian disruptions [64]. Bright light therapy has shown mixed findings in BD [58]. Accordingly, despite the promise of behavioral and biological approaches to address disrupted circadian rhythms in BD, further treatment development is needed [65].

### The applicability of the TRE to mood disorders

There is reason to think that TRE would be particularly helpful for those with BD because those with remitted BD demonstrate more irregular eating-related rhythms than individuals without psychiatric or sleep disorders [66]. Although no studies have examined TRE as a BD-specific intervention, researchers have considered mood changes in TRE trials among medical populations without diagnosable mood disorders. In the sole published RCT, depression scores significantly decreased after TRE compared to a control condition [67]. Other work has shown benefits of TRE compared to a control condition for quality of life [68, 69]. Uncontrolled studies of TRE have shown no significant decrease in pre- to post-depression scores [70, 71], although low depressive symptom levels at baseline might help explain these inconsistent findings.

We recently completed the first study to assess the effects of TRE among patients with BD. We surveyed 207 participants who self-identified as having BD and reported attempting TRE (daily food intake window of < 14 h) for at least several weeks. For each outcome assessed, 51–66% of participants reported improvements (“better” or “much better”) while implementing TRE, and 99% of participants reported gains in at least one domain, as listed in Table 1. No less benefit was apparent for those who had continued TRE for longer periods. These preliminary findings are especially promising, given that participants were trying TRE without the formal support we plan to provide.

**Table 1:** Percent of participants with BD who reported improvements when using TRE (*n* = 202–207)

Outcome	*%*
Depression	53
Mania	54
Timing of sleep	62
Ability to sleep through the night	56
Feeling rested in the morning	56
Quality of life	60
Anxiety	56
Other emotions or stress	61
Binge eating	61
Appetite or weight	55
Attention or thinking	57
Relationships with others	57
Motivation	51
Self-esteem	66

N’s vary by 1–5 due to missing data per item.

## The present project

The aim of this study is to examine the acceptability and efficacy of TRE in BD. We will examine the hypothesis that TRE leads to improvements in mood symptoms and quality of life compared to a control condition. To maximize the ability to show that TRE leads to changes in diurnal rhythms, we will recruit individuals who are (a) diagnosed with BD, (b) have at least some symptoms of circadian rhythm sleep–wake disorders or sleep disorders, and (c) report an eating window of ≥ 12 h.

Participants will be randomly assigned to TRE vs. the control intervention of the Mediterranean Diet. We chose the Mediterranean Diet as a comparison condition given that it is comparable in effort and rationale to TRE. Both TRE and the Mediterranean diet have been found to yield benefits in cardiovascular disease, diabetes, and metabolic parameters across multiple randomized controlled trials. Given the high rates of these comorbid medical conditions among those with BD and the evidence that these conditions can predict worse symptom outcomes, these are ideal healthy interventions for those with BD.

### Hypotheses

This is a hybrid trial designed to assess acceptability and efficacy.

- H1: Compared with the control intervention, TRE will lead to improvements in interview-based mania and depression scores immediately postintervention, which will manifest in downstream improvements in self-rated quality of life (QoL) at 6 months compared to baseline.
- H2: To extend findings to BD, in that TRE will be rated as highly acceptable, and patients will exhibit high adherence.
- H3: Compared with those of a control intervention, the effects of TRE will be more powerful earlier in the course of the disorder (< 5 years from onset) than later in the course of the disorder.
- H4: Compared with those of a control intervention, the effects of TRE on symptoms will be more sustained at 6, 9, and 15 months. The effects of TRE on QoL, compared to those of a control intervention, will be sustained for 6, 9, and 15 months.
- H5: Compared with a control intervention, TRE will lead to improvements in self-rated symptoms of mania and depression at the end of intervention and at 3.5, 6, 9, and 15 months after baseline compared to baseline symptom severity scores.

We will conduct intent-to-treat analyses but will conduct sensitivity analyses to consider effects among those who adhere to interventions.

## Secondary hypotheses

To examine the effects of TRE on day/night rhythms, we will assess the following effects on improving sleep hygiene behaviors (Sleep Household Environment and In-Bed Behaviors), sleep quality (Consensus Sleep Diary and Patient-Reported Outcomes Measurement Information Systems (PROMIS) self-ratings), sleep midpoint variability (Munich Chronotype Questionnaire; MCTQ), and regularity of daily routines (Brief Social Rhythm Questionnaire). To provide a more refined index of early changes in emotion, we will assess the effects of TRE on daily emotional lability (as assessed using ecological momentary assessment (EMA)). We expect adherence to both dietary interventions to be related to improvements in verbal memory (short form of the Rey Auditory Verbal Learning Test (RAVLT)). We will conduct exploratory analyses to examine a broader range of secondary outcomes and to test potential moderators.

## Method

This trial will be conducted by an international multidisciplinary team of researchers, clinicians, and lived experience experts. The study was reviewed and approved by the University of California Committee for the Protection of Human Subjects (protocol number CPHS # 2022–10-15725). The trial objectives and protocol align with all aspects of the WHO Trial Registration Data Set (version 1.3.1) and the Standard Protocol Items: Recommendations for Interventional Trials guidelines [72]. Any changes to this trial protocol will be described in publications. The findings will be reported to the scientific community according to the Consolidated Standards of Reporting Trials (CONSORT) general guidelines and the eHEALTH criteria [73]. Potential financial conflicts of interest will be reviewed annually by the University of California committee for conflicts of interest, and their guidance will be followed in managing any potential conflicts identified.

## Trial design

The trial is a prospective, rater-blind, superiority randomized controlled trial (RCT) with a one-to-one allocation ratio comparing TRE with the Mediterranean diet. Participants in both conditions will be asked to sustain ongoing medical care for BD.

The primary endpoint is immediately postintervention (at completion of the 8-week intervention). The follow-up times are 6, 9, and 15 months after the baseline, with an additional 16 weeks post-baseline self-reported assessment. The study setting is remote (online).

## Randomization

Participants will be allocated to intervention arms using a one-to-one ratio by predetermined computer-generated codes so that randomization is free of potential allocation bias. Maximally tolerated imbalance (MTI) large stick randomization will be used to match the two groups on two variables: bipolar I vs. bipolar II diagnosis and the number of hypomanic/manic episodes (coded as 1, 2, 3 +). The MTI will be set to 3. Randomization will be performed within REDCap, by a staff member not involved in assessing participants.

To reduce expectancy effects, the trial is framed as a comparison between two healthy eating interventions, and participants will not be informed of the study’s primary hypotheses. User permissions will be strictly limited, so that assessors will remain unaware of the participants’ intervention conditions. Those who become aware of the intervention condition will, if possible, be replaced. Success in masking intervention conditions will be quantified by evaluating whether interviewers are more accurate than chance when asked to guess allocation upon completion of the RCT.

## Inclusion/Exclusion criteria

The inclusion/exclusion criteria were designed to ensure the safety of the participants and the applicability of the intervention. Within these limits, we aim to gather a heterogeneous sample to test the generalizability of the results. We will recruit adults aged 18–65 years who meet diagnostic criteria for bipolar I disorder or bipolar II disorder (but not cyclothymia, BD not otherwise specified or BD due to another medical condition). We will advertise to specifically oversample those in the early stage of their illness (within 5 years of disorder onset) to be able to assess the applicability of findings for early intervention.

To improve our ability to assess whether TRE improves sleep- or circadian-related indices, the entrance criteria will include current sleep (insomnia, hypersomnolence) or circadian sleep–wake (delayed phase, advanced phase, irregular sleep–wake, non-24-h sleep–wake-type) concerns, which are reported by more than 75% of those with BD [74]. Participants who endorse at least some sleep or circadian-related impairment according to the screening self-reports or interviews will be considered to meet sleep or circadian inclusion criteria.

The other inclusion criteria included the following:

- Living in an English-speaking country (and one that we have expertise in research procedures and diet)
- Has been speaking English for at least 10 years.
- Receiving medical care for BD (referrals will be provided for those who would like to begin care)
- Mood-stabilizing medication regimens stable for at least one month (Patients with BD II whose psychiatrist sees them regularly but does not recommend ongoing mood-stabilizing medication are eligible to take part).
-  < 5 kg weight change in the past 3 months
- Currently eating ≥ 12 h per day at least twice per week
- Able to operate the camera function and respond to surveys by smartphone (loaner phones will be provided as needed)
- Able to complete 7 days of dietary logs adequately (e.g., at least 2 entries per day, covering at least a 5-h eating window) during the baseline period
- Able to complete screening and baseline questionnaires adequately (e.g., not failing more than 1 attention check item with instructed responding; responding to standard multiple-choice items in a mean of < 2 s per item). When individuals respond to more than 14 items in a row with the same response, we will manually review for possible invalidity.

The exclusion criteria include the following:

- Current episode of depression, hypomania or mania, or psychosis (assessed by the Diagnostic Interview for Anxiety, Mood, and OCD and Related Neuropsychiatric Disorders (DIAMOND)). Participants with acute mood disorder episodes will be encouraged to seek treatment and to consider the study when symptoms have remitted.
- Eating disorder diagnosis (by self-reports of treatment or diagnosis at any point during life, Short Eating Disorder Examination Questionnaire (EDE-QS) scores above clinical concern thresholds for eating disorders, or DIAMOND interview of symptoms during adulthood)
- Past 3-month alcohol use disorder or substance use disorder (assessed by the DIAMOND)
- Active suicidal ideation coupled with plan, intent or attempt history as assessed by the Columbia Suicide Severity Rating Scale (C-SSRS; [75, 76])
- Conditions that would interfere with ability to take part in the intervention, including type I diabetes, pregnancy, breastfeeding, uncorrected hypo- or hyperthyroidism, or gastrointestinal conditions impairing nutrient absorption.
- Engaged in current shift work or have other responsibilities, such as providing care that would chronically disrupt their sleep (i.e., > 3 h between 22:00 and 05:00 h for at least 1 day/week);
- Medications contraindicated for fasting; clozapine; glucose-lowering medications; diabetes-related injections; medications requiring food early in the morning or late in the evening, corticosteroids; medications such as semaglutide will not be an exclusion criteria
- Cognitive deficits as noted during the initial interview or as indicated by low performance on the Orientation Memory Concentration Test (OMC > 9)

## Recruitment and Assessment

The study will be administered at the University of California, Berkeley, with enrollment targeting individuals across English-speaking countries where we have adequate expertise, e.g., the U.S., the UK, Australia, India, and Canada. Recruitment will be open to English-speaking individuals and will be conducted primarily via social media sites (e.g., International Bipolar Foundation Facebook site, CREST-BD website, and other associations serving people with mood disorders); through websites and groups offering support for members with BD from diverse backgrounds; via general mental health sites, media, and listservs; and through traditional online and offline advertisements posted in clinical settings and community agencies. Participants will be paid $25 USD per hour for baseline and follow-up assessments and will not be paid for time spent implementing or following the dietary plan or for the screening questionnaire or screening interview.

### Online screening

Individuals interested in participating will be invited to visit the study website, where they can view the study details, view a consent form, and complete basic screening questions for medical and demographic variables (e.g., have been diagnosed with BD, aged 18–65 years, not experiencing medical conditions that would interfere with taking part in TRE; currently report eating 12 or more hours per day at least twice per week; speaking English; and living in an English-speaking country where we have adequate expertise). Individuals will be asked to complete the EDE-QS [77], and those above clinical thresholds (≥ 15) will be excluded, as will those who endorse a history of diagnosis or treatment of eating disorders. Scores above clinical thresholds on the DIAMOND Self Report Screener subscale for psychosis [78], the Alcohol Use Disorder Identification Test (AUDIT; [79]) and the Drug Use Disorder Identification Test (DUDIT; [80]) will be used to indicate who should have diagnostic interviews to assess those exclusion criteria. Questionnaires to assess sleep disturbance will include the Insomnia Severity Index (ISI; [81–83]; ≥ 8 reflective of subsyndromal insomnia), the MCTQ [84] for circadian disturbance (midpoint of sleep on free days < 4.16 h to define early chronotype and > 4.53 to define late chronotype), and irregular sleep or social activity rhythms, as indicated by a score ≥ 30 on the Brief Social Rhythm Scale (BSRS; [85]).

### Screening interview

Those who pass the online screening questions will be provided a link to schedule an appointment for an online interview to cover further eligibility criteria. The screening interview will begin with written informed consent. Then, interviewers will assess diagnoses of BD I or II and whether the potential participant is enrolled in (or willing to arrange) medical care for BD following a stable medication regimen, as well as exclusion criteria of active suicidality (CSSR) and current mood episode (DIAMOND; [78]). Those with BD II per the DIAMOND will be permitted to engage in the trial if not taking mood-stabilizing medication, as long as their provider has supported this plan and continues to see them at least once every 3 months.

We will use the Structured Clinical Interview for Sleep Disorders-Revised (SCISD-R; [86]) to assess sleep (hypersomnolence disorder) and circadian sleep–wake disorders (irregular sleep–wake disorder and non-24-h sleep–wake type disorder). We will use the DIAMOND eating disorders modules to assess symptoms since the age of 18. For those who endorse at least one relevant item on the DIAMOND-self report psychosis scale or who surpass thresholds on the AUDIT or DUDIT, we will assess current diagnoses of psychosis, alcohol use disorder, or substance use disorder. Participants who do not have a medical provider for their BD will be provided with support and referral information and invited to recontact us once they have enrolled in treatment. Participants who are in an acute episode or who are actively suicidal (assessed with the C-SSRS, discussed below) will be provided with support around engaging their provider and invited to return for follow-up assessment after one month to reconsider eligibility. Once participants provide us with their permission to contact a medical practitioner, we will send a letter to their practitioner noting that the patient has enrolled in the trial, emphasizing that we are not taking over their care and that we will potentially contact the provider for crisis management.

### Baseline assessment

Participants who meet the eligibility criteria will complete two weeks of daily logs to monitor baseline food intake and timing via a cellphone app. Enrollment in the intervention will be contingent on the adequacy of their dietary logging on the app during the baseline period. Participants will be considered to have adequate logging for a given day if they enter at least two logs at least five hours apart (they will receive information about how to add missed entries). Participants who report a much shorter time window than they did on the screening questionnaire will be reminded to capture each eating epoch, and queried if the interval logged is substantively shorter. Participants’ logging will be reviewed on the first and second days after baseline, and participants will be given the opportunity to troubleshoot with staff as needed. Thereafter, the logs will be monitored at least twice per week. Those who successfully complete logs will receive encouraging messages through the logging app. At the end of the first week, participants with no days of adequate logging will be excluded from the study (unless we identify a barrier that can be addressed). After 10 days, those who have completed less than 50% of their assigned logs will be reminded that study continuation will depend on successful logging and given an opportunity to receive support around barriers to logging; if warranted (e.g., the patient experienced technical or travel barriers), we will extend the baseline period for four additional days.

Participants who complete at least seven days of adequate dietary logs during baseline will be randomly assigned to an intervention. To allow for shopping and preparation, individuals will receive their intervention assignment 4 days before the start of the intervention, with instructions not to begin the diet until the intervention period begins.

Starting on day 5 after baseline, we will gather several daily measures sent by REDCap through text message. Participants will complete the Core Consensus Sleep Diary (Core CSD; [87]) to assess sleep quantity, timing and quality each morning for 10 days until the end of the baseline period. Participants will also complete EMA of 5 prompts per day for 8 days with brief probes to assess mood (detailed below). We will send the CSD and EMA probes on at least 2 free days for each individual (e.g., one Saturday and Sunday). After the 8 days of mood monitoring, participants will be asked to complete day-after food frequency diaries on the final two days of baseline (one free day and one workday) to capture the quantity consumed for each component of the Mediterranean Diet (e.g., fruit, vegetables, whole grains, olive oil).

After at least 3 days of adequate logging, participants will be sent links to schedule the baseline interview (longitudinal interval follow-up evaluation [LIFE]; [76, 88, 89]; Montgomery-Asberg Depression Rating Scale [MADRS]; [90]); Young Mania Rating Scale [YMRS]; [91]; and the short form of the Rey Auditory Verbal Learning Test [RAVLT]; [92, 93]) and will be asked to complete a preintervention baseline battery of online questionnaires for demographic information, symptoms, sleep, and functional information (Patient Mania Questionnaire [PMQ-9]; [94]), Patient Health Questionnaire [PHQ-9]; [95], and Brief Quality of Life in Bipolar Disorder (QoL.BD; [96]), PROMIS Sleep Disturbance and Sleep-Related Impairment [97], Sleep Household Environment and In-Bed Behaviors [98] World Health Organization Disability Assessment Schedule (WHODAS 2.0-Brief; [99]). The participants were also asked to complete measures of potential moderators (Household Food Security Survey [HFSSM]; [100]), Positive Urgency [101], Personality Traits (PID5BF + ; [102]), Risky Families [103], and for women in appropriate age ranges, the Menopause-Specific QoL Sleep [hot-flash] items [104, 105]).

### Assessments during the intervention and follow-up

Participants will be asked to complete daily food logs using brief text entries and optional photos with a cellphone app throughout the 8-week intervention period. At 6, 9, and 15 months after the end of baseline, participants will log their food (first intake and last intake) for 10 days in the app. Sleep diaries (core CSD) and EMA data will be collected for 8 days at the midpoint of the intervention. For one weekday and one weekend after the EMA ends, all participants will be asked to provide day-after food frequency diaries to be able to code Mediterranean diet adherence. Symptom severity and LIFE interview assessments will be conducted at the midpoint, at the end of the intervention, and at 6, 9, and 15 months after the end of baseline. Self-rated assessments of symptom severity, QoL, and sleep/circadian variables will be conducted at each of those assessments and at 16 weeks post baseline. The secondary outcome and eating disorder measures will be administered postintervention but not at follow-ups.

### Assessment of training and reliability

Before commencing the study, interviewers will be trained to standardize the administration and to attain high interrater reliability on all observer-rated measures (the DIAMOND, SCISD-R, C-SSRS, LIFE, MADRS and YMRS). Interrater reliability reviews will be conducted monthly on randomly selected recordings throughout the trial. If rater drift is identified, assessors will be retrained.

## Strategies to maximize data quality

As noted above, we will exclude participants who provided careless responses during the screening questionnaire. In self-rated assessments during the intervention and follow-up, we will exclude data from any bundle in which participants fail 50% or more of the attention checks [1] or when the mean duration for completion of standard multiple-choice questions is < 2 s. At the midpoint, at the end of the intervention, and at the follow-up assessments, we will ask participants whether we should exclude their data from that session due to problems with attention or understanding. Comprehensive data cleaning (range and other distributional checks, comparison with published norms where relevant) will be conducted before the analyses.

## Risk management

Risk management procedures are based on recent online self-help interventions in BD and on websites for BD [106, 107]. In this population, evidence that a participant is experiencing active suicidality would be of particular concern. All staff members will be trained to alert a licensed clinical psychologist if they learn of active suicidality (ideation coupled with intent, plan, preparation or a history of suicide attempt). As part of enrollment, we will clarify that participants will be required to continue to work with their local treatment team for their care, as we are not able to provide emergency services and do not review patient information in real time. We will require a release of information to contact a treatment provider in case of concerns about suicidality, and we will provide the participant with a letter for their provider that outlines their patient’s involvement in our study. This letter will clarify that primary treatment for the participant remains with their provider and that we may contact their provider if we are concerned about the participant’s wellbeing. At each assessment of pharmacotherapy, we will check to see if there has been a change in the provider; if so, we will gather a new release information. If we become aware of acute risk of suicidality, we will work collaboratively with patients to enhance their contact with the provider. We will inform all participants that if we are concerned about suicide risk, we may be mandated to contact their provider or authorities to intervene. We will provide resource information as part of consent (unsuicide.org, 988).

To monitor the potential development of eating disorder symptoms, we will administer the EDE-QS at the end of week 2 of the intervention, as well as at mid- and postintervention. Scores above the clinical threshold will be assessed using procedures detailed below.

### Adverse events

In addition to the risk management procedures outlined above, we will take several steps to minimize the potential for adverse events. All team members involved in clinical assessments, coaching, or otherwise in direct contact with participants will receive careful training concerning self-harm, suicidality, and elevated eating disorder, mania or depression symptom severity scores. A detailed manual will provide assessment probes, decision rules, referral resources, and contact information for use during emergency situations. Training will emphasize procedures for providing feedback in a clinically sensitive manner, and role plays will be conducted to cover legal and ethical guidelines, including situations in which we do and do not have the authority to share concerns with treatment providers and/or emergency departments. Team members will be trained about the specific situations in which we have permission to break confidentiality and contact their treatment provider or emergency services, as well as be trained in the C-SSRS Interview to assess current intent, plan, and means and history of attempts. Interviewers will be trained to contact a licensed clinical psychologist while on the phone/videoconference if any participant endorses significant suicidal ideation, and clear flow charts will be available that detail “significant suicidal ideation” (e.g., intent, plan, or means are endorsed; history of suicide attempt with current suicidal ideation).

Feedback and referrals for participants will be provided based on the judgment of urgency of the situation. In situations that do not necessitate emergency intervention, the researcher reviews the nature of the symptoms with the participant and provides support for sharing these concerns with their provider. We will encourage sharing information with providers if participants are experiencing symptoms that meet the diagnostic criteria of the DIAMOND or the LIFE for a hypomanic, manic, or depressive episode or if the MADRS score ≥ 19 [108]. More intensive interventions might involve collaborating to remove lethal means or conjointly contacting treatment providers with the participant to facilitate effective communication. As warranted, the researcher will follow-up with the participants to ensure that they were able to receive adequate support. At all times, we balance respect for autonomy with the need for safety. Participants who experience symptoms that would interfere with the demands of the protocol will be granted a break from following the food plan and logging, and interviewers will schedule a time to check in to consider restarting trial engagement.

Throughout the trial, participants will be encouraged to reach the PI with any concerns about the study. Adverse events will be assessed formally post-intervention and at each follow-up. We may also learn of problems as participants complete logs (e.g., food restriction) or interact with coaches.

All sources of information will be monitored in a timely manner to consider potential adverse events. Where clinical deterioration is noted as potentially emerging, researchers will attempt to assess whether emergent symptoms or concerns appear in any way connected to the intervention or study procedures. If EDE-QS symptoms are above the clinical threshold after enrollment, we will include a probe to assess intervention effects on those symptoms (e.g., do you believe that this intervention has triggered food or appearance symptoms for you?), and we will conduct clinical interviews to consider deterioration of symptoms. Participants will be withdrawn from the trial if their participation compromises clinical care as determined by the study’s executive committee. On a case-by-case basis, this might include instances of hospital admission, acute hypomania or mania, psychotic symptoms, active suicidal intent, or increased eating disorder symptoms, particularly where there is concern that deterioration is related to trial engagement.

Adverse events will be reviewed at monthly executive meetings and at least annual meetings of the independent trial committee (who will consider patterns of adverse events by condition). Adverse events will be promptly reported to the Committee for the Protection of Human Subjects within one week.

### Confidentiality

For all participants, we will take strong measures to protect confidentiality. Our privacy standards match those of the countries that we are recruiting in. For US participants, we have obtained an NIH Certificate of Confidentiality. All study personnel will be required to complete comprehensive training in confidentiality and privacy. When interviewing participants, we will offer meetings by secure, HIPAA-protected zoom calls; participants have the option to turn off their camera during those meetings.

Once a participant is enrolled in the study, a study ID will be assigned, and this ID will be used in place of identifying personal information such as a name on all assessment materials. The list linking the IDs and names will be destroyed after study completion. All data will be encrypted and kept on a password-protected computers that can be accessed only by study personnel. Consent forms, payment information and contact information will be filed separately from the research records. Data for this project will be gathering REDCap, a highly secure and robust web-based research data collection and management system. Features of REDCap that protect participants' privacy and data security include data storage on servers located in ITG's Advanced Computing Center providing locked physical security; use of both an OHSU firewall and a second ACC firewall; data transmissions that are encrypted with industry-standard SSL methods.

## Intervention content

We will be careful to inform participants that our study does not involve dieting and that we expect them to sustain their caloric intake throughout the intervention. Both interventions will be administered as self-help via online and app-based instruction supplemented with optional coaching sessions. The format, timing, and intensity of online and app-based coaching for the two interventions will be entirely parallel.

To minimize attrition and nonadherence, the intervention is limited to 8 weeks. Participants are welcome to continue to follow the food plan after the assigned 8 weeks and they will retain access to intervention materials. Consistent with multiple guidelines, all intervention content was reviewed by individuals with personal and family experience with BD to integrate their suggestions about the utility and refinement of the material [109, 110].

During the baseline period, we will use principles of motivational interviewing to ask participants about their own personal reasons for making changes in their food plan. We will also provide psychoeducation about “SMART” goals, including making one small change in habits at a time and avoiding self-criticism about days in which it is difficult to follow the plan. Participants will receive supportive reminders and praise for completing key components of the daily monitoring entries.

When participants are informed of their assigned intervention condition, they will receive a several-page resource guide with detailed instructions, tips, and advice for how to follow that plan (see intervention description below). Participants will receive more detail on the benefits and how to address potential barriers to the plan through brief blogs (generally one page or less) 2–3 times per week throughout the 8-week intervention period. Much of the educational content has been developed and used in previous trials of TRE and the Mediterranean Diet, but we will provide some tailored content for BD. Resource guides and blogs will be made publicly available at the end of the trial.

Because interactive support improves adherence to web interventions [111], both interventions include personal coaching support through asynchronous email contact with a trained coach, as well as opportunities to schedule a 15-min online videoconference or phone session. Participants will be given opportunities to email or to schedule 15-min sessions for coaching about barriers they face with implementing the food plan. We will encourage an initial coaching session when participants learn about their randomization and a second coaching session one week into the intervention period. At one, three, five, and seven weeks into the intervention, they will receive a link offering them a chance to email us or to schedule appointments for coaching if they would like (by clicking on a link to a calendar and specifying phone or video conference). Participants will be instructed that the scope of this coaching is narrow and will only focus on the dietary intervention. Participants can send as many messages as they like to their coach and will receive one response per week. Coaching sessions will also be limited to no more than one a week. Participants will be invited to bring significant others, family members or household members to the coaching sessions if they like.

Coaches will receive a set of guidelines and will meet with licensed clinical psychologists regularly throughout the intervention for support and to ensure that coaching maintains fidelity to the scope planned. Coaches will aim to provide a supportive, nonjudgmental environment where participants can discuss barriers and difficulties in implementing dietary plans and can receive behavioral tips. The focus of coaching will be on collaborative problem solving and empowerment in addressing barriers. Coaches will aim to help participants lessen self-criticism and to be sensitive to mood fluctuations that may make following this type of program difficult. Coaches will aim to help participants identify material in blogs, video clips, or other barriers materials that might help address the concerns raised.

Participants will also have access to a website of information designed to address barriers to following their food plan (e.g., coping with food cravings, adapting the Mediterranean Diet to culturally preferred foods), improving sleep, or addressing light exposure. Barriers were identified by reviewing previous studies using TRE or the Mediterranean diet, by conducting a survey of individuals with BD who had tried TRE, and by reviewing concerns raised by those with lived experience of BD and/or following dietary programs. For each major barrier, brief tips are available, which vary in format (written, audio, or video); all tips are designed to take 2 min or less. To help participants access relevant materials, they will be sent a quick survey at the end of weeks 3, 5, 7, and 9 in the program asking if they had experienced any of the core barriers and providing the option to view relevant tips.

### Time-Restricted eating

TRE involves restricting the window of eating to 10 h per day. To make that happen, people typically avoid eating in the first 1–2 h after they wake up and in the 2–4 h before they go to sleep. Participants will log the timing of their food intake during the baseline weeks. At the time of randomization, they will receive a graph of that data, along with a guided worksheet to help set the eating window that works best for them.

Those with an eating window greater than 14 h will be asked to restrict their eating to 12 h in the first week and then 10 h in the second week. When selecting the eating window, we will ask them to consider family meals and social commitments and will review logs from baseline to help identify those social engagements. We will also review past week sleep schedules, to plan for avoiding food 2–3 h before sleep. In setting the start time for eating in the morning, we will review any special energy demands, such as exercise, and the time periods that might work for eating given family and work commitments. Participants will be reminded not to restrict their caloric intake. As one example, people who habitually snack at night will be coached to consider ways to add calories to their regular mealtimes to avoid hunger. We will also proactively review potential barriers, such as family attitudes toward meal schedules and children’s meal timing. We also will plan ahead for upcoming holidays and vacations that may disrupt following the plan.

During the fasting period, only water, unsweetened herbal tea or black coffee, and prescription medication are permitted. During the feeding window, no restrictions are placed on the type or quantity of food consumed. We will instruct participants to follow their habitual diet within the specified consistent 10-h period and to aim to consume the same number of calories per day as they did during the baseline.

Blogs and orientation materials will provide support for keeping eating within the time window, including ways to share the food plan and goals with family and friends, ways to have easily prepared foods on-hand to avoid delays in eating dinner, and suggestions for portable breakfast ideas that would allow one to eat during a mid-morning break at work. During the third and fourth weeks of the TRE intervention, participants will receive two blogs to provide brief educational content on sleep and circadian rhythms and then two blogs to provide tips for improving sleep and circadian rhythms.

To monitor the timing of eating, participants will be asked to log each time they consume food or beverages (except water) using a cellphone app. Participant logs will be reviewed at least twice per week, and participants will be sent feedback if they appear to not log, to struggle with the time window, or to restrict the time window or caloric intake beyond the recommended goals.

### Mediterranean diet

The Mediterranean Diet is a plan for healthy eating based on how people eat and drink in the 16 countries that border the Mediterranean Sea. Individuals will be encouraged to consume vegetables (6 servings/day), fruits (2–4 servings/day), whole grains (daily), legumes (3–4 times per week), nuts (0.5 oz per day), and oily fish (2 servings/week). Participants will be encouraged to choose lean meats and other sources of protein over red meat and processed meats. Sweets (including desserts and refined cereals), alcohol, and wine or alcohol will be labeled extras, and participants will be encouraged to limit their consumption of extras. The Mediterranean diet resource kit provided at the time of randomization will include a list of key foods, a plan for setting feasible goals for change in the first week, an outline for planning grocery shopping, suggestions for following the Mediterranean diet on a budget, healthy snack options, and tips for finding recipes that meet cultural or food allergy guidelines. Interventions will be followed for eight weeks. To match the burden and self-monitoring of the TRE condition, participants in the Mediterranean diet condition will also be asked to log their food intake using the app.

## Measures

**Fig. 1:**
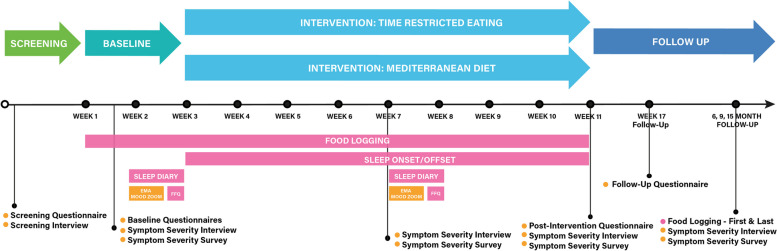
Assessment Timing

The schedule of the assessments is provided in Fig. 1 and Table 2. Assessments are performed as closely as possible to specified time points. Interviews will be conducted by HIPAA-compliant zoom or, if the participant prefers, by telephone. Participants will be sent calendar links to sign up for their interview assessments. Self-report assessments will be completed online via a secure, encrypted online survey platform (REDCap). Logs and EMA will be conducted using cellphone apps. Self-report measures and logs will be reviewed for completion and signs of carelessness, and follow-up will be conducted as warranted.

**Table 2:** Schedule of assessments

Measure	Format	Screening	Baseline	Mid-Intervention	Post-Intervention	Week 17 Follow Up	6, 9, 15 Month Follow Ups
***Screening Self-Report***							
DIAMOND Self Report Screener, Psychosis Subscale	SR	X					
Alcohol Use Disorders Identification Test (AUDIT)	SR	X					
Drug Use Disorders Identification Test (DUDIT)	SR	X					
Short Eating Disorder Examination Questionnaire (EDE-QS)	SR	X		X	X		
Insomnia Severity Index (ISI)	SR	X					
Munich ChronoType Questionnaire (MCTQ)	SR	X	X	X	X		X
Brief Social Rhythms Scale (BSRS)	SR	X	X	X	X		X
***Screening Interview***							
Orientation, Memory and Concentration Test (OMC)	I	X					
Diagnostic Interview for Anxiety, Mood, and OCD and Related Neuropsychiatric Disorders (DIAMOND)	I	X					
Columbia Suicide Severity Rating Scale (C-SSRS)	I	X					
Structured Clinical Interview for Sleep Disorders—Revised (SCISD-R)	I	X					
***Mood Assessments***							
Montgomery-Asberg Depression Rating Scale (MADRS)	I		X	X	X		X
Young Mania Rating Scale (YMRS)	I		X	X	X		X
Longitudinal Interval Follow-up Evaluation (LIFE)	I			X	X		X
Patient Mania Questionnaire (PMQ-9)			X	X	X	X	X
Patient Health Questionnaire (PHQ-9)			X	X	X	X	X
Patient Global Impression (PGI)	SR			X	X	X	X
Clinician Global Impression (CGI)	I			X	X		X
***EMA of Emotion States***							
Mood Zoom (MZ)	App		8 days	8 days			
***Quality of Life***							
Brief Quality of Life in Bipolar Disorder (QoL.BD)	SR		X	X	X		X
***Secondary Outcome Measures***							
Rey Auditory Verbal Learning Test	I		X		X		
WHO Disability Assessment Schedule 2.0-Brief	SR		X		X		
General Anxiety Disorder-7 (GAD-7)	SR		X		X		
Rapid Measurement Toolkit-20 (RMT20)	SR		X		X		
Godin Shephard Leisure-Time Physical Activity Index	SR		X		X		
***Intervention Acceptability Probes***							
Ratings of the acceptability of the Intervention	SR				X		
***Treatment Adherence***							
Food Logging	App		Daily	Daily			10 days- first & last intake
Food Frequency Questionnaire (FFQ)	SR		2 days	2 days			
Sleep Household Environment and In-Bed Behaviors	SR		X		X		
***Potential Mechanisms of Change***							
Core Consensus Sleep Diary (CORE CSD)	App		10 days	10 days			
PROMIS: Sleep Disturbance and Sleep-Related Impairment Scales	SR		X	X	X		X
***Potential Moderators***							
Personality Inventory for *DSM-5*, Brief Form Plus” (PID5BF +)	SR		X				
Positive Urgency Scale	SR		X		X		
Household Food Security Survey (HFSSM)	SR		X				
Menopause QoL items on sleep disruption (for women ages 45–65 (MENQoL)	SR		X				
Risky Families	SR		X				
Somatotherapy	SR	X	X	X	X	X	X
Demographics	SR		X				
Credibility and Expectancy Questionnaire – Credibility Set	SR		X				

Note.* App* Application, *I* Interview, *SR* Self-report

### Screening questionnaires

DIAMOND Self Report Screener [78]. We will administer the DIAMOND Self Report Screener psychosis subscale, a well-validated index of psychotic symptoms.

Alcohol Use Disorder Identification Test (AUDIT; [69, 79, 112]**).** We will administer the AUDIT to screen for potential alcohol use concerns. For individuals who score above the threshold on the AUDIT (≥ 8), we will administer the DIAMOND alcohol use disorder module to further evaluate participant eligibility.

Drug Use Disorder Identification Test (DUDIT; [80, 113]). We will administer the DUDIT to screen for potential drug use concerns. For individuals who score above the threshold on the DUDIT (≥ 6 for men; ≥ for women and nonbinary individuals), we will administer the DIAMOND substance use disorder module to further evaluate participant eligibility.

Insomnia Severity Index (ISI;[81, 83]). The ISI is a 7-item, well-validated index of nighttime and daytime insomnia symptoms**.**

Short Eating Disorder Examination Questionnaire (EDE-QS;[77]). The EDE-Q is a self-report measure designed to assess a broad range of eating-related symptoms, including restraint, eating concern, shape concern and weight concern [114]. We used the 12-item short form of the EDE-Q. This version has shown strong internal consistency (Cronbach’s α = 0.913, high temporal stability (ICC = 0.93), and scores are strongly correlated with the original EDE-Q scores (r’s > 0.81). It has also shown sensitivity in differentiating people with and without eating disorders.

Munich Chronotype Questionnaire (MCTQ; [84]). The MCTQ is designed to assess chronotype, with a focus on the midpoint of sleep during free days.

Brief Social Rhythms Scale (BSRS;[115]). The BSRS assesses the irregularity of engagement in daily activities such as mealtimes and social interaction on workdays and free days.

### Screening interviews

Orientation Memory Concentration Test (OMC;[116]). The OMC is a well-validated, brief test of mental status.

Diagnostic Interview for Anxiety, Mood, and OCD and Related Neuropsychiatric Disorders (DIAMOND; [78]). The DIAMOND is a brief structured diagnostic interview for major psychiatric disorders that has shown strong validity and reliability in comparison with other structured diagnostic interviews [78]. We will use the DIAMOND to document bipolar I or bipolar II disorder diagnoses to assess lifetime or current episodes of major depressive disorder and to ensure that we are excluding those with eating disorders (anorexia, bulimia, binge eating disorder or) at any time since age 18. Those whose symptoms on the DIAMOND are consistent with other specified eating or feeding disorders will also be excluded (e.g., repetitive binging or purging that does not meet the frequency or duration criteria). For those with scores above the threshold on the AUDIT (≥ 8) or DUDIT (≥ 6 for men; ≥ 2 for women and nonbinary individuals), we will administer the alcohol use disorder and/or substance use disorder modules as appropriate to consider exclusion criteria. For those who endorse any symptoms of psychosis on the DIAMOND-self report, we will complete the psychosis module of the DIAMOND.

Columbia Suicide Severity Rating Scale (C-SSRS; [75]). The C-SSRS is a brief, commonly used scale designed to cover suicidal ideation and behavior. Moreover, the use of other measures of suicide risk has been well validated. We will assess suicidal ideation, intent, plan and means in the past 2 weeks and lifetime history of suicide attempt using the screening version of the C-SSRS.

Structured Clinical Interview for Sleep Disorders—Revised (SCISD-R; [86]). The SCISD-R is a semi-structured clinical interview used to assess common sleep disorders. We will use the SCISD-R to assess hypersomnolence disorder and circadian sleep–wake disorders.

## Outcome measures

### Mood assessments

Our primary measures of mood will be interview-based assessments. We will include self-reported measures of mood in addition, as these allow for more frequent and affordable assessments. We will enrich our understanding of the temporal dynamics of change in these processes with our daily measures of mood (EMA), which will be gathered at baseline and at the midpoint of intervention. For comparison with the findings of other studies, we will also gather the Clinical Global Impression (CGI) and Patient Global Impression (PGI) indices. Our primary outcome measures will be interview-based measures of mood symptoms: the MADRS, YMRS, and LIFE score.

Montgomery-Asberg Depression Rating Scale (MADRS;[117]). The MADRS is a brief, widely used, psychometrically sound measure designed to briefly assess the severity of depressive symptoms. It provides standardized probes and behavioral anchors and attains high interrater reliability across studies of unipolar and bipolar disorder.

Young Mania Rating Scale (YMRS;[91]). The YMRS is one of the most frequently used rating scales for assessing the severity of manic symptoms. Each of the 11 items covers the severity of a core symptom of mania. The ratings reflect participant responses to the interview, coupled with clinician observation.

Longitudinal Interval Follow-Up Evaluation (LIFE; [76, 118]). We will use the LIFE to gather variations in manic mood states across the period between assessments to potentially enrich our ability to “catch” small changes between follow-ups. The LIFE will include probes for rapidly cycling symptoms [115].

We will also include the Wellcome Trust recommended self-report measures for depression and mania, the Patient Health Questionnaire (PMQ-9 for mania and PHQ-9 for depression; [94, 95]. Both are brief scales that have been shown to have high internal consistency and to be sensitive to treatment effects.

### EMA of emotion states

We will supplement our gold standard symptom severity interviews with ecological momentary assessment (EMA) probes of emotion states. An EMA has the advantage of providing a potentially more sensitive indicator of early shifts in mood variability [119]. Participants will be sent 5 surveys per day on their smartphones. Participants will be randomly assigned to one of four schedules (adapted to their designated time zone). These schedules allow for three hours between the final survey of the day and their usual bedtime. Surveys will be quasi-randomized during those hours, spaced on average 135 min apart. Participants will have up to 80 min to respond to each survey and will receive one reminder if they do not respond within the first 40 min.

In our EMA, we will use the Mood Zoom probes, which have been validated in BD [120], with additional probes for sleepiness, hyperfocus, and feeling inspired using a parallel format. Our core index derived from dimension reduction of the emotion probes above is the mean variability in daily negative affect (mean square of successive difference). As a secondary test of proposed mechanisms, we will also assess whether TRE, as a circadian intervention, leads to decreased evening levels of arousal (using factor scores). As a secondary outcome, we predict that both indices will decline more from baseline to the intervention midpoint in the TRE condition than in the Mediterranean diet condition and that this level of decrease will predict greater change in symptom severity and quality of life.

### Quality of life

We will use the Brief Quality of Life in Bipolar Disorder (QoL.BD) questionnaire as our index of quality of life [121]. QoL. BD was designed to capture a full spectrum of relevant domains for individuals with BD (physical, sleep, mood, cognition, leisure, social, spirituality, finance, household, self-esteem, independence, and identity) and to be sensitive and pragmatic for repeated assessments in the context of treatment. The measure was developed based on previously available measures, with consultation from consumers and international experts in BD. It showed strong internal consistency and correlations with objective measures of functioning [122]. This short version is based on factor-analytic methods, has been validated for online use, and has been shown to be sensitive to change in two RCTs [123, 124].

## Secondary outcome measures

In addition to the secondary mood outcomes noted above, we will gather data to assess several other secondary outcomes.

Rey Auditory Verbal Learning Test (RAVLT;[92, 93, 125]). We will administer the RAVLT to assess cognitive benefits of both interventions, given that both interventions are expected to improve inflammation [126–131] and that inflammation has been shown to be related to cognitive performance for those with BD [132–134]. This test has been shown to be sensitive to cognitive deficits associated with BD, even during euthymic periods [135, 136]. We will use the short form that has been adopted by the NIMH Toolkit.

To harmonize our findings with those of other Wellcome Trust grants, we included three measures as secondary outcome measures: the World Health Organization Disability Assessment Schedule 2.0—Brief (WHODAS 2.0-Brief; [99])**,** GAD-7 [137] and the panic, social anxiety and PTSD subscales of the Rapid Measurement Toolkit (RMT20; [138]). The WHODAS 2.0-Brief is a 12-item survey that assesses overall role functioning across major life domains. The GAD-7 is a commonly used measure of generalized anxiety symptoms. The RMT20 is a brief screening tool developed using IRT analyses and well validated against clinical diagnoses [138].

### Intervention acceptability probes

We will use acceptability probes previously validated in the ORBIT clinical trial for BD [109), rated on a scale from “Strongly Disagree” to “Strongly Agree”. We will supplement these with specific questions to assess whether individuals perceive that their mania, depression, sleep, or quality of life improve or worsen following the intervention. Our primary index of acceptability will be the percentage of individuals who endorse that they agree or strongly agree that they would recommend the food plan to a friend. Ratings of “agree” or “strongly agree” for each item will be considered acceptable. As a further check of potential negative outcomes, participants will be asked open-ended queries regarding problems associated with the intervention. For transparency, we will present a table with the full set of items for evaluating acceptability.

### Treatment adherence

#### Food intake schedules

 Food intake schedules, including adherence to the planned schedule, will be assessed using reminders sent by cellphone. The logs are designed to be simple to complete and to lower burden by allowing participants to take a photo of their food or to briefly note the food without the burdens of inputting portion size or calories. Participants can complete their diet log in real time, although missed entries can be added retroactively. Researchers have full access to the data, which will never be shared with commercial entities. This type of photo logging of food by cellphone logs has been validated in several ways [139]. First, compared with reports from questionnaires covering longer time periods, the number of ingestion events captured through a cellphone app is comparable or surpasses the number of meal records found in NHANES 24-h recall data. Second, the caloric content reported surpasses calculated energy requirements. Third, logs capture a significantly longer duration of eating windows per day and more variability in eating rhythms (including weekend delays in breakfast).

Adherence will be scored based on the time of their first and final caloric consumption each day. We will use well-validated metrics for scoring this based on previous research with TRE, in which we will define the time interval that contains 95% of intake events. This approach is less skewed by occasional nonreporting of events or infrequent deviations from the diet and thus provides a more robust and reliable approach to measuring change [140]. Following standards in other US and European studies of TRE, we will focus on days in which participants adequately logged (e.g., entered at least two intake events, covering at least a 5-h window) and will calculate the percentage of days in which individuals met the eating window goal. High adherence will be defined as meeting this standard on at least 78% of the days logged. As supplemental data, we will report the percentage of days logged and the percentage of days in which individuals logged adequately and followed the planned window.

#### Mediterranean diet adherence

Adherence to the Mediterranean diet will be assessed using a food frequency questionnaire (FFQ) gathered at baseline and at the midpoint of intervention. We designed FFQ probes to score the Alternate Mediterranean Diet score, which is an updated version of the original Mediterranean Diet score to integrate median intake levels by sex [141, 142]. Within this scoring system, participants receive 1 point for the consumption of > median amounts of vegetables, fruits, legumes, nuts, whole grains, and fish; for a favorable ratio of monounsaturated to saturated fats; and for lower levels of red or processed meats and for lower alcohol use. These FFQ diaries will be supplemented with coding based on the text or photo food log entries.

### Potential mechanisms of change

To determine whether TRE specifically shifts sleep and day/night rhythms (hypothesized mechanisms), we will examine changes in sleep (the gold standard CORE CSD measures of quality of sleep and reduced variability in sleep midpoint, along with PROMIS sleep disturbance and sleep-related impairment) and day/night rhythms (BSRS and the MCTQ). To assess whether the sleep and light guidance offered as part of the TRE intervention leads to improved sleep hygiene, we will gather the Sleep Household Environment and In-Bed Behaviors [98].

### Potential moderators

We chose to include participants taking a broad range of medications to enhance the generalizability of the sample. To examine how medication levels shape intervention effects on circadian and symptom outcomes, we will gather the types of psychotropic and sleep medicines, doses, and adherence levels at each self-report assessment to calculate summary scores for each receptor type. At baseline, we will gather data on self-rated personality symptoms using the Personality Inventory for DSM-5, the Brief Form Plus (PID5BF + ; [102]), the Positive Urgency Scale [143], food insecurity (HFSSM; [100, 144]), Menopause QoL sleep items [104, 105], and early adversity (the Risky Families Scale; [145]) as potential predictors of intervention outcome.

## Reasons for discontinuation from study

Participants requesting to withdraw from the study will be asked to complete an intervention acceptability survey and final assessment of symptoms (self-rated mania, depression, and QoL; and, if willing, the YMRS, MADRS, and the LIFE. They will be asked (via a brief phone call from a trial coordinator) the main reasons for discontinuation. Should adverse events be discovered, those will be reported.

## Statistical analysis plan

We chose our sample size to support showing a moderate effect size for changes in symptoms in the TRE vs. control conditions (f2 = 0.25). We planned for 33% attrition based on previous online intervention studies in BD, to support a sample size of 200. A sample size of 200 provides > 80% power (1-*β*) to detect an effect of this size at α = 0.05 (two-tailed) and to detect changes in TRE compared to those of the Mediterranean diet within those with early onset (estimated to be one-third of the sample).

Before conducting the analyses, we will examine whether the assumptions for missing value imputation are met (e.g., whether values appear to be missing at random). If those assumptions are supported, we will impute data using 20 resamples, using demographic (gender, age), baseline symptom severity, and symptom history variables (number of episodes) to predict missing scores. For comparison, we will perform analyses without imputed values.

### Data cleaning

In addition to reviewing careless responses during screening, we will review each self-report bundle for careless responding. We will qualitatively review and consider excluding questionnaire bundles where a person has responded with the same response more than 14 times in a row or has a high Mahalanobis distance score.

### Statistical analysis of the primary outcome

Changes in mania, depression, and QoL from baseline to immediately postintervention are hypothesized to be significantly greater in the TRE intervention than in the Mediterranean diet intervention. We will test this using a multivariate repeated measures linear mixed model analysis with random effects. All intervention-related effects will be estimated using an intention-to-treat approach, in which we will examine final scores (where available) for all randomized participants. To support intent-to-treat analyses, we will attempt to gather final assessments for all participants who discontinue the study (except those who explicitly discontinued or were withdrawn on ethical grounds). For H2, we will provide descriptive information, and we will test the hypothesis that relative to the Mediterranean Diet, the TRE condition will be associated with decreased attrition and higher ratings of satisfaction with the intervention. Statistical analyses will be conducted in accordance with the International Conference on Harmonization E9 statistical principles.

### Sensitivity analysis of the primary outcome

To corroborate the robustness of the findings, we will conduct analyses restricted to those participants who completed the baseline and postintervention symptom assessments and who also showed at least 80% adherence to the intervention during the last month of the intervention (early weeks are expected to include growth in the ability to follow both food plans). For TRE, we will define the eating window as the time window during which 95% of the eating events (the 2.5% to 97.5% interval) occur, and then calculate the proportion of days in which the person followed the planned time window. For the Mediterranean Diet, adherence will be quantified as a mean score of 6 or higher (roughly the 90th percentile) on the Alternate Mediterranean Diet score for the two days assessed at the midpoint of intervention [146].

### Statistical analyses of secondary outcome measures

Intervention-related changes in secondary outcomes will be tested using repeated measures linear mixed model analyses to determine whether TRE leads to changes in sleep (core CSD, PROMIS Sleep Disturbance and Sleep-related Impairment), day/night rhythms (BSRS and the MCTQ), or emotion variability (as assessed using ecological momentary assessment) as compared to the Mediterranean Diet.

## Discussion

This study will provide novel evidence about the benefits of TRE relative to the likely beneficial effects of the Mediterranean diet on symptoms and QoL in BD. If TRE has a greater effect than does the Mediterranean diet, the intervention could have considerable public health implications, as the intervention is free for patients and easily implemented, and an app to support the intervention can be offered worldwide. Although our primary targets are BD symptoms and QoL, the high prevalence of comorbid medical conditions observed in BD patients necessitate considering broader approaches that could enhance psychiatric and medical health. The present study then sets the stage for potential dissemination of a web-based tool for TRE that could have multiple benefits for those with BD. The study also provides novel and important data regarding the benefits of the Mediterranean diet, which one might expect to reduce metabolic diseases that are all too profoundly correlated with BD.

Despite the potential public health advantages of this approach, the current study has several key limitations. First, the study was not designed to carefully assess the circadian processes believed to underlie benefits or to assess benefits for metabolic conditions. Parallel research is needed to examine these proposed mechanisms, as the international, large-scale nature of this study prevents more intensive biological assessment. Second, our goal here is to examine this as a potentially low-cost, easily disseminated intervention. It remains possible that even if TRE or the Mediterranean diet are helpful, more intensive interpersonal interaction and coaching could be required for people to be able to implement the plan.

We hope that the current study will provide important information about the applicability of dietary interventions for patients with BD. If the findings are positive, the current research will establish the basis for rapid dissemination of this approach in the community.

## Data Availability

After the data collection has been completed, de-identified data, data analysis scripts, statistical analysis plan, informed consent, publicly available (non-copyrighted) measures will be shared on the Open Science Foundation wtihin one year after data collection ends, where it will be available upon request. We will also comply with Wellcome Trust guidance for data sharing.
